# NK1‐receptor‐expressing paraventricular nucleus neurones modulate daily variation in heart rate and stress‐induced changes in heart rate variability

**DOI:** 10.14814/phy2.12207

**Published:** 2014-12-03

**Authors:** Claire H. Feetham, Richard Barrett‐Jolley

**Affiliations:** 1 Institute of Ageing and Chronic Disease, Centre for Integrative Mammalian Biology, University of Liverpool, Liverpool, UK

**Keywords:** cardiovascular, paraventricular nucleus, substance P

## Abstract

The paraventricular nucleus of the hypothalamus (PVN) is an established center of cardiovascular control, receiving projections from other nuclei of the hypothalamus such as the dorsomedial hypothalamus and the suprachiasmatic nucleus. The PVN contains a population of “pre‐autonomic neurones” which project to the intermediolateralis of the spinal cord and increase sympathetic activity, blood pressure, and heart rate. These spinally projecting neurones express a number of membrane receptors including GABA and substance P NK1 receptors. Activation of NK1‐expressing neurones increases heart rate, blood pressure, and sympathetic activity. However, their role in the pattern of overall cardiovascular control remains unknown. In this work, we use specific saporin lesion of NK1‐expressing PVN rat neurones with SSP‐SAP and telemetrically measure resting heart rate and heart rate variability (HRV) parameters in response to mild psychological stress. The HRV parameter “low frequency/high frequency ratio” is often used as an indicator of sympathetic activity and is significantly increased with psychological stress in control rats (0.84 ± 0.14 to 2.02 ± 0.15; *P* < 0.001; *n* = 3). We find the stress‐induced increase in this parameter to be blunted in the SSP‐SAP‐lesioned rats (0.83 ± 0.09 to 0.93 ± 0.21; *P* > 0.05; *n* = 3). We also find a shift in daily variation of heart rate rhythm and conclude that NK1‐expressing PVN neurones are involved with coupling of the cardiovascular system to daily heart rate variation and the sympathetic response to psychological stress.

## Abstract (precis)

The paraventricular nucleus of the hypothalamus contains NK1‐receptor‐expressing neurones hypothesized to influence cardiovascular control and the cardiovascular response to stress. In this work, we show by HRV analysis increases in LF/HF ratio in response to psychological stress, consistent with an increase in sympathetic activity. Lesioning NK1‐receptor‐expressing neurones in the PVN abolished this response and resulted in a 3‐h shift in the daily variation of heart rate.

## Introduction

A population of paraventricular nucleus (PVN) hypothalamic parvocellular neurones projects directly to sympathetic control “centres” of the medulla and spinal cord (Pyner and Coote 2000) and modulates heart rate (HR) and blood pressure (BP) (Coote 2007). The activity of these neurones becomes elevated in heart failure as their tonic inhibitory GABA‐ergic input becomes reduced (Pyner 2014). Although this pathway is therefore of huge importance to cardiovascular medicine, there is no consensus as to its specific role in cardiovascular control. Theories to date include mediation of the cardiovascular response to stress, control of blood volume, and circadian changes in HR. In our previous work, we have shown that these neurones can be controlled by tachykinin neuropeptides (Womack et al. 2007). In this work, we report the effect of selective lesion of PVN neurokinin 1 (NK1)‐receptor‐expressing neurones on heart rate and heart rate response to psychological stress in rats.

A number of neurotransmitters and modulators are known to act on spinally projecting neurones, including GABA, glutamate, nitric oxide, and adenosine (Pyner 2009; Nunn et al. 2011; Affleck et al. 2012). However, recent focus has been on the tachykinin family of neuropeptides (including substance P, SP), since evidence suggests that the tachykinins (including SP), especially NK1 receptor activating ligands (Culman and Unger 1995; Culman et al. 2010; Tauer et al. 2012), are important for the central control of mean arterial blood pressure (Culman and Unger 1995; Culman et al. 2010). In our own recent work, we characterized, in vitro, an SP‐dependent pathway linking the PVN to another important cardiovascular control center in the hypothalamus; the dorsomedial hypothalamus (DMH) (Womack and Barrett‐Jolley 2007), and an associated SP‐activated (sympathostimulatory) pathway projecting from the PVN to the intermediolateral spinal cord (Womack et al. 2007).

The PVN has been known to be a site for integration of the hormonal response to stress (Herman and Cullinan 1997) for some time, and it was recently confirmed that a proportion of the noxious stress response (subcutaneous formalin) is sensitive to intracerebroventricular (ICV) application of a selective NK1 and NK2 antagonist (Culman et al. 2010). Furthermore, psychological stress (using elevated plus maze test) is markedly reduced in rats given ICV injection of a selective NK1 receptor antagonist. In the same study, stress‐induced c‐Fos expression within the PVN is lower after pharmacological blockade of the NK1 receptor (Ebner et al. 2008). Reduced c‐Fos expression in the PVN is also seen in NK1R^−/−^ mice subjected to same stressor (Santarelli et al. 2002). Levels of the stress hormone cortisol are decreased compared to their wild‐type counterparts as a result of this stress test (Santarelli et al. 2001). However, the theory that the PVN is generally important for the cardiovascular response to stress (Dayas et al. 2004) remains controversial. While stimulation of the PVN modifies BP and HR (Kannan et al. 1989; Martin et al. 1991, 1993; Duan et al. 1997; Schlenker et al. 2001); others maintain that the PVN is not involved with the cardiovascular response to stress itself (Stotz‐Potter et al. 1996; Fontes et al. 2001; DiMicco et al. 2002). One possible explanation for this is that since “stress” is a term which describes a wide range of physiological and psychological stimuli, certain forms of stress (such as subcutaneous formalin, (Culman et al. 2010)) may activate tachykinin‐mediated PVN responses, whereas others do not. It is also possible that tachykininergic spinally projecting neurones may mediate other facets of cardiovascular control. For example, PVN “pre‐sympathetic” neurones have been implicated circadian control of BP (Cui et al. 2001).

In this work, we use a specific saporin lesion of NK1‐expressing PVN rat neurones with substance P‐saporin (SSP‐SAP) and measure resting heart rate and heart rate variability (HRV) parameters in response to mild psychological stress. We detected no change in overall daytime heart rate, or in heart rate response to stress, but we find changes in daily heart rate rhythm and HRV response to psychological stress. The HRV parameter “low frequency to high frequency ratio (LF/HF)” is often used as an indicator of sympathetic activity and significantly increased with psychological stress. We find the stress‐induced increase in this parameter to be blunted in the SSP‐SAP lesion rats. We conclude that NK1‐expressing PVN neurones are involved with both the coupling of the cardiovascular system to daily variations in heart rate and the sympathetic response to psychological stress.

## Methods

### Ethical approval

All animal work was carried out in accordance with the UK Animals (Scientific Procedures) Act 1986 under a Home Office Licence. All surgery was performed under general anesthesia as described in detail below.

### Animals

All procedures were performed on young adult male Wistar rats (200–400 g; *n* = 6). Rats were maintained in the animal facility of the University of Liverpool on a 12–12 h light–dark cycle. All animals had unlimited access to water and standard chow diet.

### Immunofluorescence

Rats were terminally anesthetized by intraperitoneal injection of pentobarbitone (Pentoject, Animalcare, York, UK; 60 mg·kg^−1^) and perfused transcardially with 4% paraformaldehyde in PBS. Tissues were then removed and dehydrated with 30% sucrose in PBS overnight at 4°C and 14 *μ*m coronal cryostat sections prepared (Leica, Milton Keynes, UK). Immunofluorescence was performed using the primary antibody anti‐rabbit Neurokinin‐1 receptor (1:500; Abcam, Cambridge, UK) combined with the secondary antibody donkey anti‐rabbit Dylight 594 (1:2000; Abcam, UK), and finally DAPI nuclei staining (0.1 *μ*g·mL^−1^; Invitrogen, Paisley, UK). Cell counts were performed and efficacy of lesion was confirmed to be 100%.

### Paraventricular nucleus of the hypothalamus‐targeted injections of SSP‐SAP

Specific lesions of the entire PVN were performed by the injection of the cytotoxic Substance P‐saporin (SSP‐SAP) (0.04 mg·mL^−1^; Advanced Targeting Systems, San Diego, USA); a conjugation of saporin and SSP, the Sar^9^, Met(O_2_)^11^ analog of Substance P, shown to be selective in many studies (Khasabov and Simone 2013; Talman and Lin 2013).

**Figure 1.**
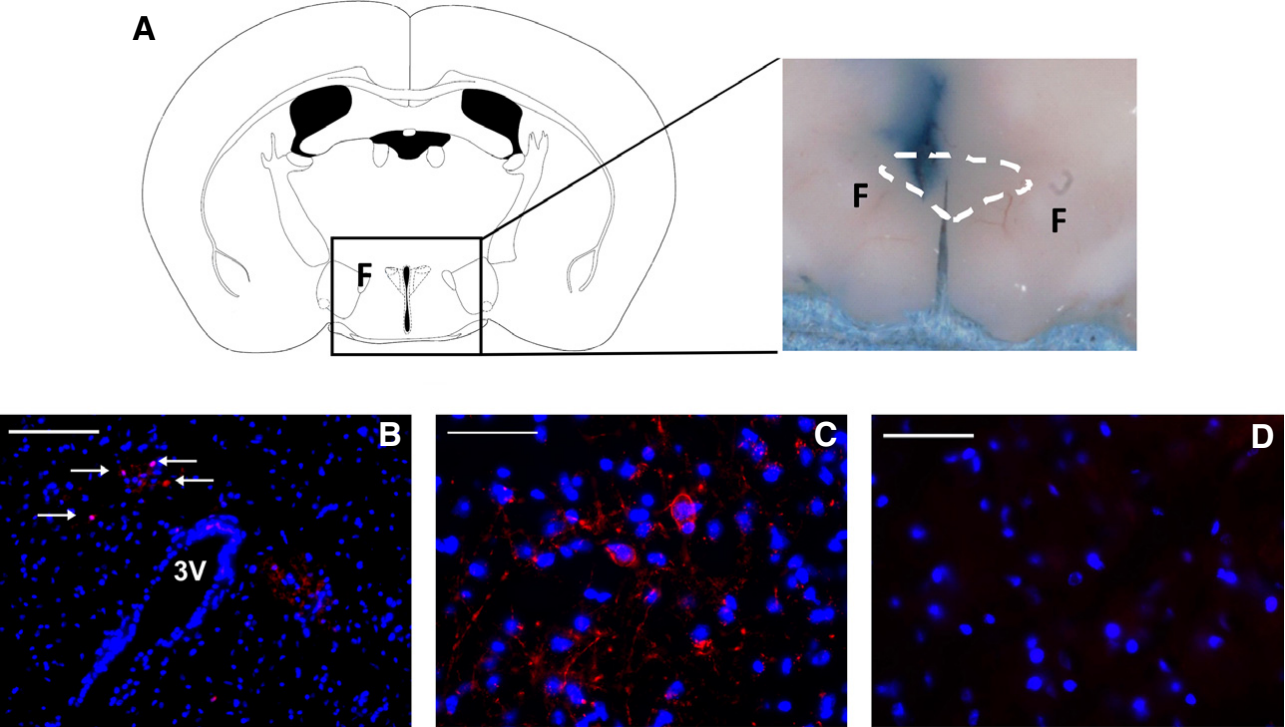
Selective lesion of NK1‐expressing neurones in rat PVN. (A) Unilateral injection with pontamine blue (1%). F=fornix. The dotted line indicates the approximate position of the PVN. Note that no dye crosses to the contralateral side. (B) Low magnification image of coronal section of PVN showing orientation using the third ventricle. Intact side (left of the third ventricle) and lesioned side (right of the third ventricle), showing clear red staining for the NK1 receptor using the primary antibody anti‐rabbit Neurokinin‐1 receptor (1:500; Abcam, UK) combined with the secondary antibody donkey anti‐rabbit Dylight 594 (1:2000; Abcam, UK) and blue DAPI nuclei staining (white arrows indicate staining). Scale bar is 100 *μ*m (C) Intact side of the PVN used as a positive control. Scale bar is 50 *μ*m (D) Lesioned side of the PVN from the same Wistar rat shows an absence of red NK1 receptor staining; blue DAPI nuclei staining remains. Scale bar is 50 *μ*m.

Prior to surgery, adult male Wistar rats (*n* = 6; 200–400 g) were put under isoflurane gas anesthesia (4% v/v induction; 2% v/v maintenance) surgery was performed under aseptic conditions. Preoperative subcutaneous injections of the analgesic buprenorphine (Temgesic, 1.5 mg·kg^−1^; Reckitt Benckiser, Slough, UK), the antibiotic enrofloxacin (Baytril, 0.2 mL·kg^−1^; Bayer AG, Leverkusen, Germany), and the anti‐inflammatory meloxicam (Metacam, 100 *μ*g·kg^−1^; Boehringer Ingelheim, Berkshire, UK) were given. 50 nL SSP‐SAP (*n* = 3) or 50 nL PBS (control; *n* = 3) was injected unilaterally in the right hand side gradually over a few minutes via a 5 *μ*L Hamilton syringe at previously defined PVN coordinates (1.8 mm caudal, 1.8 mm lateral, 9.2 mm vertical at an angle of 10°). These injections sites were based on the rat atlas and adjusted according to the size of the rat. Site specificity was confirmed using immunofluorescence and dye injections (Fig. 1) (Paxinos and Watson 1986). The Hamilton syringe was left in the injection site for 5–10 min to avoid residual solution moving up the track from the syringe as much as possible.

### Telemetry surgery, recording, and mild stress handling

During lesion surgery, electrocardiogram transmitters (ETA‐F20; Data Sciences International, St Paul, MN) were also implanted subcutaneously into rats under isoflurane gas anesthesia. The rats were monitored postoperatively, and were allowed at least 7 days of recovery before any further procedures began. This recovery period was found to be sufficient for the reestablishment of normal HR patterns (Thireau et al. 2008) and for the lesion to take effect. Rats were housed individually over receiver pads (Data Sciences International) and ECG recorded continuously. The ECG signal was digitized to a PC with a CED Micro1401 using Spike2 at 5 kHz. Heart rate was annotated using a custom program. Mild stress was induced by handling of the rats (Balcombe et al. 2004) a few days after recording began.

### HRV analysis

Heart rate variability analysis was performed using the Kubios HRV program (Niskanen et al. 2004). For power spectrum analysis, HR was resampled at 20 Hz, and 3‐min sections of clean and stable HR were analyzed by fast Fourier transform using Welch's periodogram with 50% overlapping windows of 32 sec. Low‐frequency (LF) and high‐frequency (HF) bandings were 0.15–1.0 and 1.0–5.0 Hz, respectively (previously verified by (Nunn et al. 2013)).

### Statistics

Data were analyzed by one‐way ANOVA unless otherwise stated (Minitab). All data are presented as means ± SEM. Power equations: we assumed a 6% SD of heart rate (Nunn et al. 2013) and effect size of 20%. A statistical power of 80% (*α *= 0.05) required two groups of three animals.

## Results

### Efficacy of lesion

To confirm the action of the SSP‐SAP lesion and the coordinates we have derived based on the stereotaxic rat atlas (Paxinos and Watson 1986) we used immunofluorescence of the NK1 receptor on the PVN. As the lesion was unilateral the side which remained intact was used as a positive control. Figure 1A shows the intact side of the PVN, red staining indicates NK1 receptor staining, DAPI nuclear staining is blue. Figure 1B clearly shows the SSP‐SAP‐lesioned side of the PVN; the lesion resulting in an absence of red NK1 receptor staining.

### Effects of PVN NK1 lesion on 24‐h heart rate

**Figure 2.**
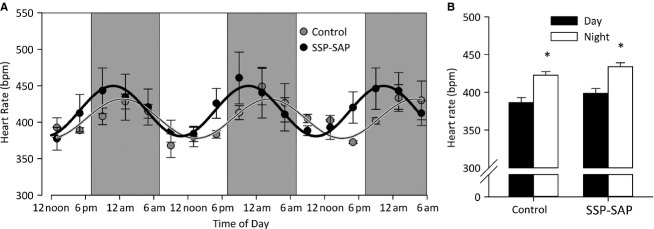
Daily variation in heart rate in SAP‐SSP lesioned rats. (A) Circadian variation in heart rate was plotted as average heart rate per 4 h in both control and SSP‐SAP‐lesioned rats. These data were fit with a standard sigmoidal waveform and a significant shift in the circadian phase from 3.28 ± 0.16 to 4.49 ± 0.20 radians was observed (*P* < 0.05 Student's paired *t*‐test). (B) Control and lesioned rats both show increased heart rate at night compared to during the day; from 387 ± 6 to 423 ± 5 beats min^−1^ in control (*n* = 3; *P* < 0.001 by one‐way ANOVA) and 399 ± 6 to 436 ± 5 beats min^−1^ in lesioned rats (*n* = 3; *P* < 0.001 by one‐way ANOVA). No differences between the two groups were observed.

ECG was obtained in freely moving conscious rats using subcutaneous implantation of telemetric transmitters, and heart rate data were derived using a custom program. Daily variation in heart rate was plotted as average per 4 h. Both control and lesioned animals showed increased heart rate at night compared to during the day (Fig. 2); from 387 ± 6 to 423 ± 5 beats min^−1^ in control (*P* < 0.001 by one‐way ANOVA; *n* = 3 per group) and 399 ± 6 to 436 ± 5 beats min^−1^ in lesioned rats (*P* < 0.001 by one‐way ANOVA; *n* = 3 per group).

These data were fit with a standard sigmoidal waveform: 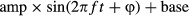


Where amp is the amplitude in bpm (i.e., the difference between maximum nighttime and minimum daytime heart rate), *f* is the frequency in h^−1^ (defined as 1/24), *φ* is the phase in radians, and base is the baseline heart rate. There was a significant shift in the heart rate phase from 3.28 ± 0.16 to 4.49 ± 0.20 radians (*P* < 0.05 Student's paired *t*‐test Fig. 2B); equivalent to a 3‐h shift in the cycle.

### Effect of lesion on cardiovascular response to psychological stress

**Figure 3.**
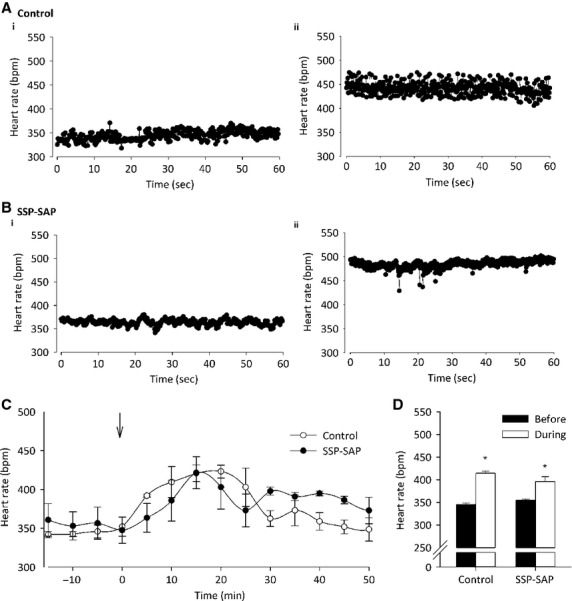
Heart rate response to stress in SAP‐SSP‐lesioned rats. (A) Raw basal heart rate traces of control rats (i) before and (ii) after mild handling stress. (B) Raw basal heart rate traces of SSP‐SAP rats (i) before and (ii) after mild handling stress. (C) Average heart rate per 5 min in both groups of rats. Arrow indicates time of mild handling stress. (D) Heart rate significantly increases both in control rats from 345 ± 2 to 414 ± 5 beats min^−1^ (*n* = 3 per group; *P* < 0.001 by one‐way ANOVA) and in lesioned rats from 354 ± 3 to 396 ± 11 beats min^−1^ (*P* < 0.05 by one‐way ANOVA;* n* = 3 per group). No difference in heart rate response to stress between the two groups was observed.

To determine the effect of mild psychological stress on cardiovascular parameters of NK1 receptor PVN‐lesioned rats, the animals were subjected to mild handling stress. Activity as little as moving a cage has been shown to increase heart rate and levels of the stress hormone corticosterone in the plasma of rats (Seggie and Brown 1975). Upon handling stress, heart rate was seen to significantly increase in a similar fashion in both control and lesioned rats (Figs. 3A, 3B, C and D); 345 ± 2 beats min^−1^ to 414 ± 5 beats min^−1^ in control (*P* < 0.001 by one‐way ANOVA; *n* = 3 per group) and 354 ± 3 beats min^−1^ to 396 ± 11 beats min^−1^ in lesioned rats (*P* < 0.05 by one‐way ANOVA; *n* = 3 per group). No significant difference in heart rate response to stress between the two groups was observed (Fig. 3D).

**Figure 4.**
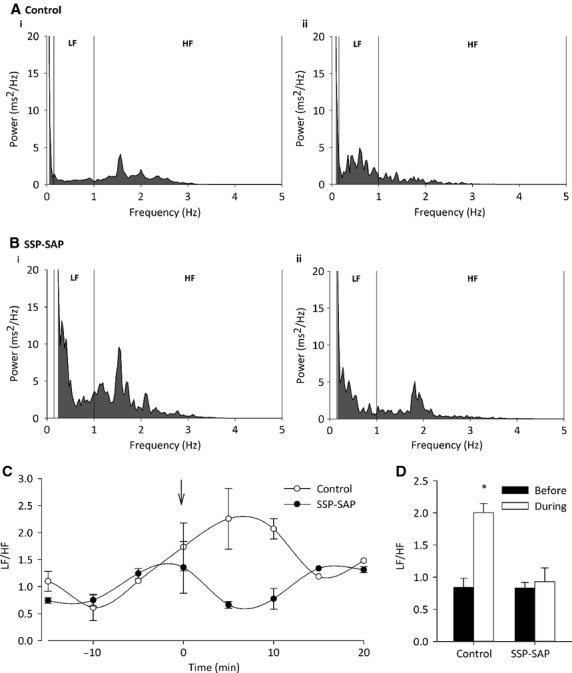
LF/HF response to stress in SAP‐SSP‐lesioned rats. (A) Representative fast Fourier transform for control rats (i) before and (ii) after mild handling stress. (B) Representative fast Fourier transform for SSP‐SAP rats (i) before and (ii) after mild handling stress. In control animals, an increase in LF and decrease in HF power are seen as a result of stress. Both LF and HF are reduced in lesioned rats after stress. (C) Average LF/HF ratio per 5 min in both groups of rats. Arrow indicates time of mild handling stress. (D) LF/HF ratio significantly increases in control animals subjected to stress from 0.84 ± 0.14 to 2.02 ± 0.15 (*n* = 3 per group; *P* < 0.05 by one‐way ANOVA). This response was abolished in SSP‐SAP‐lesioned rats (*n* = 3 per group; *P* > 0.05 by one‐way ANOVA).

HRV analysis was performed on ECG recordings, as HRV is an indication of autonomic balance. The LF‐to‐HF ratio (LF/HF) in particular, is a useful indicator of sympathetic *versus* parasympathetic balance. Using power spectra analysis, LF/HF was determined using previously validated frequency banding (Nunn et al. 2013) (Fig. 4A and B). LF/HF was significantly increased in control rats from 0.84 ± 0.14 to 2.02 ± 0.15 (Fig. 4C and D; *P* < 0.05 by one‐way ANOVA; *n* = 3 per group); indicating an increase in sympathetic activity. This response was ablated in the SSP‐SAP‐lesioned rats (Fig. 4C and D; *P* > 0.05 by one‐way ANOVA; *n* = 3 per group), suggesting a reduction in sympathetic drive due to a loss of the NK1‐expressing neurones.

## Discussion

In this work, we show for the first time that PVN NK1‐expressing neurones are involved with the daily variation of heart rate and also the sympathetic component of the response to mild psychological stress. Interestingly, the changes observed occurred after only unilateral lesion of the NK1‐receptor‐expressing neurones of the PVN. One may have expected compensation from the intact side to have nullified the effects of unilateral lesion. Two clear possibilities are (1) that the lesioning agent spread to the other side; however, this does not seem to be the case. We found that NK1 neurones were still present in the untreated side. An alternative hypothesis (2) is that the effect would indeed have been much greater if both sides had been treated. For the present experiments, we treated one side only, partly so the intact side could act as an immunofluorescent control for the treated side, in terms of NK1 neurone ablation, and partly because we were unsure as to what effect this treatment would have on the animals. Bilateral lesion may be a useful protocol to explore in future investigations of the role of NK1 receptors in the PVN.

A number of studies show conclusively that the PVN is important to cardiovascular control (Badoer et al. 2002; Coote 2005; Ramchandra et al. 2013) and although others show the PVN to be central to the HPA component of the stress response (Herman and Cullinan 1997; Herman et al. 2002; Tavares et al. 2009), the evidence that the PVN is directly involved in the sympathetic and cardiovascular stress response is less strong. Our own previous work shows that the spinally projecting “pre‐autonomic” sympathetic PVN neurones express SP receptors and that these modulate the cardiovascular system (Womack et al. 2007). Their mechanism of action is quite complex. SP interacts with the resting (tonic) inhibition of spinally projecting neurones by GABA (Womack et al. 2007). This scheme involves change of the kinetic properties of spinally projecting neurone GABA_A_ receptors and is thus, presumably allosteric. Furthermore, this cross‐talk is PKC dependent (Yamada and Akasu 1996).

One of the first studies to investigate the role of SP in cardiovascular response to stress used a combination of global NK1 knock‐out and a selective, but blood–brain barrier crossing, antagonist (intravascular) in mice. While there was a clear reduction in heart rate increase to a noxious stimulus, it was not possible to determine where the active NK1 receptors were. Elevated plus maze experiments also showed a marked decrease in the behavioral attributes of stress when rats were given a specific NK1 receptor antagonist via ICV injection (Ebner et al. 2008). While this does not identify the location of the relevant NK1 receptors, this stressor also resulted in reduced c‐Fos expression within the PVN of those rats treated with the NK1 receptor antagonist, implicating PVN NK1 receptors. Recent work by Culman et al. (2010) has also shown that ICV injection of specific tachykinin antagonists reduces the cardiovascular (and hormonal) response to stress, again these receptors could be anywhere accessible to the ICV injection. However, to investigate this further, Culman et al. (2010) analyzed the c‐Fos response of PVN neurones in response to stress with and without tachykinin antagonist. They found that the c‐Fos response of corticotropin‐releasing factor‐expressing PVN neurones was blunted by the tachykinin antagonists. This combination of studies therefore shows that NK1 receptors are involved with the cardiovascular and behavioral responses to severe (noxious) and psychological stress, and that NK1 receptors mediate at least a component of the response of PVN neurones by stress. However, we have now added one of the final pieces of data to this story by showing that reduction of NK1‐expressing PVN neurones (by SSP‐SAP unilateral lesion) mediates the LF/HF response to mild psychological stress. This type of heart rate variability analysis is often used as a method for quantifying the autonomic influence on the cardiovascular system based on HR variation over time. These natural rhythms occur at different frequencies associated with sympathetic and parasympathetic nervous system influences. HRV is therefore widely used as an accurate indicator of autonomic balance (Malpas 2002; Baudrie et al. 2007; Thireau et al. 2008) and autonomic response to stress (Farah et al. 2006). Although there is no direct HRV indicator of sympathetic activity, a number of studies, including our own (Nunn et al. 2013), have shown that the LF/HF ratio is a valid measure of autonomic balance and therefore it is possible to infer changes in sympathetic activity using this parameter (Katoh et al. 2002; Nunn et al. 2013). In our previous study, we methodically verified bandings for LF/HF boundaries and showed that atropine reduced the HF spectrum power and reserpine reduced the LF/HF ratio (Nunn et al. 2013). Furthermore, in a previous study, we directly showed that sympathetic activity of anesthetized rats was stimulated by SP (Womack et al. 2007) in anesthetized rats. We are therefore confident that our observed reduction of LF/HF power in freely moving rats does indeed indicate a genuine reduction of sympathetic activity.

We also found that PVN NK1 neurones are involved with setting the daily variation of the rats' heart rate. Since the rats were kept under a 12‐h light/12‐h dark cycle regimen, this could involve a changed behavioral response to conditions or it could suggest the involvement of these neurones in setting circadian cycles. Further experiments under fixed light conditions would be necessary to confirm the inherent hypothalamic rhythmicity has been affected rather than response to light itself. However, spinally projecting neurones of the PVN are involved with circadian rhythm. This was first suggested by Cui et al.( 2001) who showed that spinally projecting neurones received input from the suprachiasmatic nucleus; a key center of the hypothalamus involved with circadian rhythm (Reppert and Weaver 2002). Neurones in this area have cyclically changing membrane potentials which allow general changes in activity on a 24‐h rhythm (Belle et al. 2009). Studies show that this is paralleled by changes in rodent heart rate (Nunn et al. 2013) and we find that this involves PVN NK1 neurones, since their lesion significantly alters the rhythm, shifting it by approximately 3 h. This is potentially of huge medical relevance, since in humans, hypertension is strongly linked to sympathetic activity (Mancia and Grassi 2014) and circadian variation in cardiovascular control is strongly linked to a spate of heart attacks that occurs in the morning (Muller et al. 1989; Spielberg et al. 1996; Lefer 2010).

Our current data therefore provide urgently required data to show as directly as possible that the stress‐induced in sympathetic activity does involve PVN NK1 receptors and raises the possibility that potentially, selective inhibition of spinally projecting neurones could be therapeutically useful for modulation of stress‐related heart disease.

## Conflicts of Interest

The authors confirm there are no conflicts of interest.

## References

[b1] Affleck V. S. Coote J. H. Pyner S. 2012 The projection and synaptic organisation of NTS afferent connections with presympathetic neurons, GABA and nNOS neurons in the paraventricular nucleus of the hypothalamus. Neuroscience; 219:48-61. 22698695 10.1016/j.neuroscience.2012.05.070 PMC3409377

[b2] Badoer E. Ng C. W. De Matteo R. 2002 Tonic sympathoinhibition arising from the hypothalamic PVN in the conscious rabbit. Brain Res.; 947:17-24. 12144848 10.1016/s0006-8993(02)02901-3

[b3] Balcombe J. P. Barnard N. D. Sandusky C. 2004 Laboratory routines cause animal stress. Contemp. Top. Lab. Anim. Sci.; 43:42-51. 15669134

[b4] Baudrie V. Laude D. Elghozi J. L. 2007 Optimal frequency ranges for extracting information on cardiovascular autonomic control from the blood pressure and pulse interval spectrograms in mice. Am. J. Physiol. Regul. Integr. Comp. Physiol.; 292:R904-R912. 17038438 10.1152/ajpregu.00488.2006

[b5] Belle M. D. Diekman C. O. Forger D. B. Piggins H. D. 2009 Daily electrical silencing in the mammalian circadian clock. Science (New York, N.Y.); 326:281-284. 10.1126/science.1169657 19815775

[b6] Coote J. H. 2005 A role for the paraventricular nucleus of the hypothalamus in the autonomic control of heart and kidney. Exp. Physiol.; 90:169-173. 15604110 10.1113/expphysiol.2004.029041

[b7] Coote J. H. 2007 Landmarks in understanding the central nervous control of the cardiovascular system. Exp. Physiol.; 92:3-18. 17030558 10.1113/expphysiol.2006.035378

[b8] Cui L. N. Coderre E. Renaud L. P. 2001 Glutamate and GABA mediate suprachiasmatic nucleus inputs to spinal‐projecting paraventricular neurons. Am. J. Physiol. Regul. Integr. Comp. Physiol.; 281:R1283-R1289. 11557637 10.1152/ajpregu.2001.281.4.R1283

[b9] Culman J. Unger T. 1995 Central tachykinins: mediators of defence reaction and stress reactions. Can. J. Physiol. Pharmacol.; 73:885-891. 8846426 10.1139/y95-122

[b10] Culman J. Das G. Ohlendorf C. Haass M. Maser‐Gluth C. Zuhayra M. 2010 Blockade of tachykinin NK1/NK2 receptors in the brain attenuates the activation of corticotrophin‐releasing hormone neurones in the hypothalamic paraventricular nucleus and the sympathoadrenal and pituitary‐adrenal responses to formalin‐induced pain in the rat. J. Neuroendocrinol.; 22:467-476. 20210847 10.1111/j.1365-2826.2010.01987.x

[b11] Dayas C. V. Buller K. M. Day T. A. 2004 Hypothalamic paraventricular nucleus neurons regulate medullary catecholamine cell responses to restraint stress. J. Comp. Neurol.; 478:22-34. 15334647 10.1002/cne.20259

[b12] DiMicco J. A. Samuels B. C. Zaretskaia M. V. Zaretsky D. V. 2002 The dorsomedial hypothalamus and the response to stress: part renaissance, part revolution. Pharmacol. Biochem. Behav.; 71:469-480. 11830181 10.1016/s0091-3057(01)00689-x

[b13] Duan Y. F. Winters R. McCabe P. M. Green E. J. Huang Y. Schneiderman N. 1997 Cardiorespiratory components of defense reaction elicited from paraventricular nucleus. Physiol. Behav.; 61:325-330. 9035265 10.1016/s0031-9384(96)00410-6

[b14] Ebner K. Muigg P. Singewald G. Singewald N. 2008 Substance P in stress and anxiety: NK‐1 receptor antagonism interacts with key brain areas of the stress circuitry. Ann. N. Y. Acad. Sci.; 1144:61-73. 19076365 10.1196/annals.1418.018

[b15] Farah V. M. Joaquim L. F. Morris M. 2006 Stress cardiovascular/autonomic interactions in mice. Physiol. Behav.; 89:569-575. 16962148 10.1016/j.physbeh.2006.07.015

[b16] Fontes M. A. P. Tagawa T. Polson J. W. Cavanagh S. J. Dampney R. A. L. 2001 Descending pathways mediating cardiovascular response from dorsomedial hypothalamic nucleus. Am. J. Physiol. Heart Circ. Physiol.; 280:H2891-H2901. 11356650 10.1152/ajpheart.2001.280.6.H2891

[b17] Herman J. P. Cullinan W. E. 1997 Neurocircuitry of stress: central control of the hypothalamo‐pituitary‐adrenocortical axis. Trends Neurosci.; 20:78-84. 9023876 10.1016/s0166-2236(96)10069-2

[b18] Herman J. P. Cullinan W. E. Ziegler D. R. Tasker J. G. 2002 Role of the paraventricular nucleus microenvironment in stress integration. Eur. J. Neuorsci.; 16:381-385. 10.1046/j.1460-9568.2002.02133.x 12193178

[b19] Kannan H. Hayashida Y. Yamashita H. 1989 Increase in sympathetic outflow by paraventricular nucleus stimulation in awake rats 1. Am. J. Physiol.; 256:R1325-R1330. 2567578 10.1152/ajpregu.1989.256.6.R1325

[b20] Katoh K. Nomura M. Nakaya Y. Iga A. Nada T. Hiasa A. 2002 Autonomic nervous activity before and after eradication of Helicobacter pylori in patients with chronic duodenal ulcer. Aliment. Pharmacol. Ther.; 16Suppl 2:180-186. 11966539 10.1046/j.1365-2036.16.s2.27.x

[b21] Khasabov S. G. Simone D. A. 2013 Loss of neurons in rostral ventromedial medulla that express neurokinin‐1 receptors decreases the development of hyperalgesia. Neuroscience; 250:151-165. 23831426 10.1016/j.neuroscience.2013.06.057 PMC3769426

[b22] Lefer D. J. 2010 Is there a better time of day to have a heart attack? Circ. Res.; 106:430-431. 20167939 10.1161/CIRCRESAHA.109.213652

[b23] Malpas S. C. 2002 Neural influences on cardiovascular variability: possibilities and pitfalls. Am. J. Physiol. Heart Circ. Physiol.; 282:H6-H20. 11748042 10.1152/ajpheart.2002.282.1.H6

[b24] Mancia G. Grassi G. 2014 The autonomic nervous system and hypertension. Circ. Res.; 114:1804-1814. 24855203 10.1161/CIRCRESAHA.114.302524

[b25] Martin D. S. Segura T. Haywood J. R. 1991 Cardiovascular responses to bicuculline in the paraventricular nucleus of the rat. Hypertension; 18:48-55. 1860711 10.1161/01.hyp.18.1.48

[b26] Martin D. S. Haywood J. R. Thornhill J. A. 1993 Stimulation of the hypothalamic paraventricular nucleus causes systemic venoconstriction. Brain Res.; 604:318-324. 8457859 10.1016/0006-8993(93)90383-x

[b27] Muller J. E. Tofler G. H. Stone P. H. 1989 Circadian variation and triggers of onset of acute cardiovascular disease. Circulation; 79:733-743. 2647318 10.1161/01.cir.79.4.733

[b28] Niskanen J. P. Tarvainen M. P. Ranta‐Aho P. O. Karjalainen P. A. 2004 Software for advanced HRV analysis. Comput. Methods Programs Biomed.; 76:73-81. 15313543 10.1016/j.cmpb.2004.03.004

[b29] Nunn N. Womack M. Dart C. Barrett‐Jolley R. 2011 Function and pharmacology of spinally‐projecting sympathetic pre‐autonomic neurones in the paraventricular nucleus of the hypothalamus. Curr. Neuropharmacol.; 9:262-277. 22131936 10.2174/157015911795596531 PMC3131718

[b30] Nunn N. Feetham C. H. Martin J. Barrett‐Jolley R. Plagge A. 2013 Elevated blood pressure, heart rate and body temperature in mice lacking the XLalphas protein of the Gnas locus is due to increased sympathetic tone. Exp. Physiol.; 98:1432-1445. 23748904 10.1113/expphysiol.2013.073064 PMC4223506

[b31] Paxinos G. Watson C. 1986 The rat brain in stereotaxic coordinates London Academic Press Inc 10.1016/0165-0270(80)90021-7 6110810

[b32] Pyner S. 2009 Neurochemistry of the paraventricular nucleus of the hypothalamus: implications for cardiovascular regulation. J. Chem. Neuroanat.; 38:197-208. 19778682 10.1016/j.jchemneu.2009.03.005

[b33] Pyner S. 2014 The paraventricular nucleus and heart failure. Exp. Physiol.; 99:332-339. 24317407 10.1113/expphysiol.2013.072678

[b34] Pyner S. Coote J. H. 2000 Identification of branching paraventricular neurons of the hypothalamus that project to the rostroventrolateral medulla and spinal cord. Neuroscience; 100:549-556. 11098118 10.1016/s0306-4522(00)00283-9

[b35] Ramchandra R. Hood S. G. Frithiof R. McKinley M. J. May C. N. 2013 The role of the paraventricular nucleus of the hypothalamus in the regulation of cardiac and renal sympathetic nerve activity in conscious normal and heart failure sheep. J. Physiol.; 591:93-107. 22615431 10.1113/jphysiol.2012.236059 PMC3630774

[b36] Reppert S. M. Weaver D. R. 2002 Coordination of circadian timing in mammals. Nature; 418:935-941. 12198538 10.1038/nature00965

[b37] Santarelli L. Gobbi G. Debs P. C. Sibille E. T. Blier P. Hen R. 2001 Genetic and pharmacological disruption of neurokinin 1 receptor function decreases anxiety‐related behaviors and increases serotonergic function. Proc. Natl Acad. Sci. USA; 98:1912-1917. 11172050 10.1073/pnas.041596398 PMC29356

[b38] Santarelli L. Gobbi G. Blier P. Hen R. 2002 Behavioral and physiologic effects of genetic or pharmacologic inactivation of the substance P receptor (NK1). J. Clin. Psychiatry; 63Suppl 11:11-17. 12562138

[b39] Schlenker E. Barnes L. Hansen S. Martin D. 2001 Cardiorespiratory and metabolic responses to injection of bicuculline into the hypothalamic paraventricular nucleus (PVN) of conscious rats. Brain Res.; 895:33-40. 11259757 10.1016/s0006-8993(01)02011-x

[b40] Seggie J. A. Brown G. M. 1975 Stress response patterns of plasma corticosterone, prolactin, and growth hormone in the rat, following handling or exposure to novel environment. Can. J. Physiol. Pharmacol.; 53:629-637. 1175088 10.1139/y75-087

[b41] Spielberg C. Falkenhahn D. Willich S. N. Wegscheider K. Voller H. 1996 Circadian, day‐of‐week, and seasonal variability in myocardial infarction: comparison between working and retired patients. Am. Heart J.; 132:579-585. 8800028 10.1016/s0002-8703(96)90241-0

[b42] Stotz‐Potter E. H. Morin S. M. DiMicco J. A. 1996 Effect of microinjection of muscimol into the dorsomedial or paraventricular hypothalamic nucleus on air stress‐induced neuroendocrine and cardiovascular changes in rats. Brain Res.; 742:219-224. 9117398 10.1016/s0006-8993(96)01011-6

[b43] Talman W. T. Lin L. H. 2013 Sudden death following selective neuronal lesions in the rat nucleus tractus solitarii. Auton Neurosci.; 175:9-16. 23245583 10.1016/j.autneu.2012.11.008 PMC4337398

[b44] Tauer U. Zhao Y. Hunt S. P. Culman J. 2012 Are biological actions of neurokinin A in the adult brain mediated by a cross‐talk between the NK1 and NK2 receptors? Neuropharmacology; 63:958-965. 22771977 10.1016/j.neuropharm.2012.06.041

[b45] Tavares R. F. Pelosi G. G. Correa F. M. 2009 The paraventricular nucleus of the hypothalamus is involved in cardiovascular responses to acute restraint stress in rats. Stress; 12:178-185. 18609300 10.1080/10253890802246659

[b46] Thireau J. Poisson D. Zhang B. L. Gillet L. Le Pecheur M. Andres C. 2008 Increased heart rate variability in mice overexpressing the Cu/Zn superoxide dismutase. Free Radic. Biol. Med.; 45:396-403. 18513493 10.1016/j.freeradbiomed.2008.04.020

[b47] Womack M. D. Barrett‐Jolley R. 2007 Activation of paraventricular nucleus neurones by the dorsomedial hypothalamus via a tachykinin pathway in rats. Exp. Physiol.; 92:671-676. 17468202 10.1113/expphysiol.2007.037457

[b48] Womack M. D. Morris R. Gent T. C. Barrett‐Jolley R. 2007 Substance P targets sympathetic control neurons in the paraventricular nucleus. Circ. Res.; 100:1650-1658. 17495222 10.1161/CIRCRESAHA.107.153494

[b49] Yamada K. Akasu T. 1996 Substance P suppresses GABA(A) receptor function via protein kinase C in primary sensory neurones of bullfrogs. J. Physiol. (Lond.); 496:439-449. 8910228 10.1113/jphysiol.1996.sp021697 PMC1160889

